# An optimized cognitive biomarker that accurately predicts future cognitive decline and improves the detection of MCI and early dementia

**DOI:** 10.1002/alz70857_107696

**Published:** 2025-12-26

**Authors:** Maurice Smith, Daniel Z. Press

**Affiliations:** ^1^ Harvard University School of Enginering and Applied Sciences, Cambridge, MA, USA; ^2^ Berenson‐Allen Center for Noninvasive Brain Stimulation, Beth Israel Deaconess Medical Center and Harvard Medical School, Boston, MA, USA; ^3^ Department of Neurology, Harvard Medical School, Boston, MA, USA; ^4^ Beth Israel Deaconess Medical Center, Boston, MA, USA

## Abstract

**Background:**

Mild Cognitive Impairment (MCI) affects over 12 million individuals in the US, 50% of whom will progress to Alzheimer's disease or another form of dementia in 3‐5 years. But 90% of individuals with MCI remain undiagnosed due to challenges in screening. With the advent of disease‐modifying therapies (DMTs) that can slow progression, it is now critical that new and improved tools become available to quickly and accurately screen for MCI, and especially for the subpopulation of individuals at highest risk for developing future dementia.

**Methods:**

Here we use data‐driven design to create a novel tool for cognitive assessment that combines information‐efficient test‐items identified by analysis of the National Alzheimer's Coordinating Center (NACC) NIH Uniform Data Set version 3 (UDSv3) study items in > 10,000 CN, MCI, and early dementia individuals. We assessed the ability to detect MCI measured by the receiver operating characteristic (ROC) area under the curve (AUC) values, selected the best‐performing items, and determined the optimal weighting between them.

**Results:**

Using both data‐driven item selection and optimal item weighting allowed for the creation of a 4‐item brief optimized cognitive composite (BOCC) test with a 4‐5 minute administration time that can dramatically outperform the 10‐14 minute MoCA at detecting both MCI and mild dementia. We find AUC values for the BOCC that are 35% closer to the ideal 1.00 value for BOCC compared to MoCA scores for detecting MCI (0.87 vs 0.80, *p* <0.0001) and 80% closer to ideal for BOCC vs MoCA scores for detecting mild dementia (0.99 vs 0.95, *p* <0.0001). Remarkably, the BOCC score also provides more information for predicting 6‐year future conversion to dementia for MCI and CN individuals than the dementia specialists’ clinical diagnoses (AUCs: 0.95 vs 0.88, *p* <0.0001).

**Conclusion:**

A 4‐5 minute brief optimized cognitive test can dramatically outperform the 10‐14 minute MoCA in detecting MCI, and it can predict future decline to dementia comparably to, or better than, a clinical diagnosis made by clinicians with access to extensive cognitive and clinical information. This test could improve MCI detection in the more than 52 million people in the US age 65 or over.

